# Cost of illness of oral lichen planus: a multicenter university hospital–based outpatient observational study

**DOI:** 10.1007/s00784-021-04353-1

**Published:** 2022-01-10

**Authors:** Lajolo C, Rupe C, Gioco G, Giuliani M, Contaldo M, Salo T, Siponen M

**Affiliations:** 1 Head and Neck Department, “Fondazione Policlinico Universitario A. Gemelli – IRCCS”, School of Dentistry, Università Cattolica del Sacro Cuore, Largo A. Gemelli, 8, 00168 Rome, Italy; 2 Department of Clinical and Experimental Medicine, University of Foggia, Via Rovelli 50, 71122 Foggia, Italy; 3 Multidisciplinary Department of Medical-Surgical and Dental Specialties, University of Campania Luigi Vanvitelli, Via Luigi de Crecchio, 6, 80138 Naples, Italy; 4 Cancer and Translational Medicine Research Unit, University of Oulu, 90220 Oulu, Finland; 5 Institute of Dentistry, University of Eastern Finland, P.O. Box 1627, 70211 Kuopio Campus, Kuopio Finland; 6 Department of Oral and Maxillofacial Diseases and Oral Health Teaching Clinic, Kuopio University Hospital, Kuopio, Finland

**Keywords:** Cost of illness, Oral lichen planus, Oral medicine

## Abstract

**Objectives:**

To estimate the economic costs of oral lichen planus (OLP) through a multicenter university hospital–based outpatient study conducted in Italy and Finland.

**Materials and methods:**

A multicenter retrospective study was conducted on patients affected by OLP to evaluate the economic cost of managing the disease. Direct costs concerning diagnostic procedures, therapeutic management, and follow-up visits were obtained from clinical records. Statistics was performed with IBM SPSS Statistics.

**Results:**

One hundred and eight patients with a confirmed diagnosis of OLP (81 women and 27 men), 58 Italians and 50 Finnish, were enrolled in this study. The mean annual cost was 1087.2 euros per patient. The mean annual cost was higher in Finnish than in Italian cohort (1558.7 euros vs. 680.7 euros—*p* < 0.05). Within the Italian cohort, the local immunosuppressive therapy group and atrophic and erosive OLP type had a higher cost (*p* < 0.05). Within the Finnish cohort, the local immunosuppressive therapy group had a higher cost (*p* < 0.05).

**Conclusions:**

OLP-related costs are very similar to other chronic oral disorders (i.e., periodontitis) with differences between investigated countries. Moreover, patients with more severe clinical features, who need immunosuppressive therapy, are facing more expensive costs.

**Clinical relevance.:**

In this multicenter cost of illness study, we estimated the direct health care costs of OLP and we found that patients with more severe clinical features, who therefore need symptomatic therapy, are facing more expensive costs.

## Introduction

Chronic diseases are the leading cause of death and disability in the developed world of the twenty-first century [1]. They are a large group of diseases, characterized by a prolonged course, which do not resolve spontaneously and for which a complete cure is rarely achieved. Heart disease, stroke, cancer, diabetes and chronic respiratory diseases, mental illness, musculoskeletal and gastrointestinal disorders, sight and hearing defects, and genetic diseases represent some of them. These disorders significantly impact the quality of life of patients and cause a significant increase in costs related to the management of patients’ diseases and comorbidities.

Cost of illness (COI) studies allow to quantify the economic weight of a given disease through the identification, the estimate, and the sum of the relative costs in a defined period of time (i.e., prevalence-based approach) or for the entire duration of the disease (i.e., incidence-based approach) [2]. COI studies specifically identify three types of costs related to the disease: direct, indirect, and intangible costs. Direct costs represent the health care costs related to the prevention, diagnosis, and treatment of the disease (e.g., drugs, diagnostic tests, laboratory tests, medical examinations, hospitalizations, and therapies support) and non-health care costs related to goods and services utilized (e.g., expenses for the transport of patients, assistance costs incurred by the family, expenses incurred for particular dietary regimes). Indirect costs typically originate from lost work productivity (e.g., loss of working days by the patient or by those who take care of him/her) or from the loss of social well-being due to the disease. Eventually, intangible costs are the expression of a psychological and subjective dimension of the disease and, therefore, difficult to evaluate and quantify (e.g., pain, loss of quality of life, depression). This type of study, therefore, estimates not only the quantities of resources (in economic terms) used to treat the disease, but also the economic consequences due to a loss of productivity caused by the disease itself. Having a global overview of a certain condition makes it possible to define the size of the disease burden in monetary terms, and also provides support for health care planning related to disease prevention and control, as well as feedback for the evaluation of the program adopted by the public health care system of different countries [2, 3]. Particularly, Italian and Finnish public health care systems are similar, providing preventive, diagnostic, and therapeutic procedures completely free or “co-paid” by the patient: Italy provides health care service through the regional health care system, whereas Finland through local municipalities.

Lichen planus (LP) is one of the most frequent chronic disorders of the oral cavity; it is a chronic, inflammatory, immune-mediated disease of unknown etiology that can affect not only the mucous membranes of the oral cavity (oral LP), but also the skin and its adnexa (cutaneous LP), as well as the lining tissues of the external genital systems (genital LP) [4]. Oral lichen planus (OLP) occurs in the world population with an incidence ranging from 0.5 to 2%, showing a greater preference for middle-aged and women subjects. OLP has several clinical manifestations: the lesions are classified as reticular (papular), plaque-like, atrophic (erythematous), erosive or ulcerative, and bullous; often, there is the co-presence of hyperkeratotic and erythematous aspects. In particular, the atrophic and the erosive forms of OLP are associated with symptoms that vary from mild to severe. Furthermore, OLP is considered an oral potentially malignant disorder, with a risk of malignant transformation of about 1.4% [5]. Therefore, it requires a strict follow-up, and control biopsies are needed in cases with atypical clinical presentations or symptoms [6]. The chronic course of this disease, associated with a wide spectrum of clinical manifestations and symptoms, and the risk of malignant transformation require careful and meticulous management of this condition and regular follow-up. Specifically, as OLP is considered an immune-based disease that mimics a type IV hypersensitivity reaction, the main therapeutic regimen consists of local immunosuppressive drugs, mainly corticosteroids, generally administered in symptomatic patients or when inflammation is widespread [7]. These are primarily used topically, but also systemically in the most severe cases, requiring careful control of the onset of possible side effects (e.g., candidiasis, hirsutism, hypertension, hyperglycemia, Cushing’s syndrome); nevertheless, some patients do not improve with conventional local immunosuppressive therapy (non-responders) and need secondary-line therapy [8].

The purpose of this multicenter study is to evaluate the direct health care costs related to the clinical management of patients with OLP, through a retrospective outpatient university hospital–based study conducted in Finland and Italy. In particular, the impact of therapy on costs will be evaluated and differences between Italian and Finnish systems will be evaluated.

## Materials and methods

A multicenter retrospective university hospital–based observational study was conducted on outpatients with clinical and histological diagnosis of OLP, to evaluate the economic costs of the disease. Patients were referred by general practitioners (GPs) or by dentists to an oral medicine specialist at the Head and Neck Department, “Fondazione Policlinico Universitario A. Gemelli—IRCCS” (Rome, Italy) and at the Oral and Maxillofacial Diseases Clinic, Kuopio University Hospital (Kuopio, Finland). The study design was approved by the Ethical Committee of the “Fondazione Policlinico Universitario A. Gemelli—IRCCS” (Prot. 41,666/17, ID: 1729) and the Northern Ostrobothnian Hospital District (Prot. 46/2013), according to guidelines for good clinical practice of European Union and to the Helsinki Declaration. The Research Ethics Committee of the Northern Savo Hospital District was notified of the approval and Kuopio University Hospital granted an organization permit for the study.

### Patient selection and data collection

The medical records of OLP patients under treatment from 01.01.2008 to 01.08.2020 at Oral and Maxillofacial Diseases Clinic, Kuopio University Hospital (Kuopio, Finland), and from 01.01.2005 to 01.08.2020 at the Head and Neck Department, “Fondazione Policlinico Universitario A. Gemelli—IRCCS” (Rome, Italy), were revised. Only patients with clinical and histological diagnosis of OLP, according to modified WHO diagnostic criteria [9], were included in the study. Data concerning demographic characteristics (i.e., age, gender, country), clinical features (i.e., clinical type and site of OLP), diagnostic procedures (i.e., biopsies, swabs, blood exams), therapeutic (either local or systemic pharmacological therapy), and follow-up management (i.e., number of visits, follow-up) were extracted from clinical records and were collected into an ad hoc database.

### Cost analysis

The cost analysis was carried out retrospectively, considering the health care costs incurred by the patient and by the local health care system office, from the time of diagnosis and during the entire observational period. Specifically, only direct health care costs were considered: (1) outpatient costs related to diagnostic procedures and checkups (e.g., visits, biopsies, swabs), (2) costs related to therapy, both local and systemic. The information regarding the outpatient services performed (i.e., number of visits, biopsies, and swabs) and any prescribed drugs (i.e., type of drug and dosage) were obtained from the patient’s medical records. The costs of outpatient services have been estimated by referring to the fees adopted in 2018 at the Kuopio University Hospital and the Fondazione Policlinico A. Gemelli Hospital in Rome, while the costs related to pharmacological therapy have been estimated by referring to the national pharmaceutical handbook (Italy) and to the Common European Drug Database (CEDD) (Finland). The cost analysis was carried out using the bottom-up approach: the outpatient costs were calculated by multiplying the cost related to the single procedure by the number of procedures used by the patient and then they were added up, to get the total cost of each patient. The total cost was then divided by the follow-up time and the average was calculated, so as to have the average annual cost per patient. This calculation included first examination, follow-up examinations, biopsies, and swabs. To calculate costs associated with the local therapy, we first estimated the number of applications/administrations that can be carried out with a single pack, dividing the total amount of gel in a single pack by the presumed amount of gel needed for the single application. We then calculated the number of packs purchased by each patient, knowing the total number of applications administered during follow-up period. Eventually, to get the average annual cost per patient, we multiplied the cost of a single package by the total number of packages purchased by the patient, divided by the follow-up time. The same calculation was made for systemic therapy (corticosteroids, other immunosuppressants, and antimycotic). To calculate the total annual average cost per patient, the cost of services was then added, so as to have the total average annual cost.

### Statistical analysis

Sample size was calculated considering an approximal annual cost of an OLP patient without therapy of 500€ and an increase of at least 100€ for the local immunosuppressive therapy: setting type I error = 0.05 and statistic power at 95%, the total simple size was 54. Simple size calculation was performed with GPower software ver. 3.1 for Apple.

Quantitative variables were tested for normal distribution by a Kolmogorov–Smirnov test. Differences between Italian and Finnish study population and between patients undergoing local immunosuppressive therapy and those that did not receive local immunosuppressive therapy were compared: parametric variables were tested by means of two-tailed analysis of variance (ANOVA), whereas the Mann–Whitney test was used for non-parametric variables. Binomial or discontinuous variables were assessed by means of the chi-square test and Fisher’s exact test. Statistics was performed with IBM SPSS Statistics ver. 25 for Apple (IBM Corp., Armonk, NY).

## Results

One hundred and eight patients (81 women and 27 men, mean age 60.8) with a confirmed diagnosis of OLP were included in this multicenter study (58 Italians and 50 Finns) with a mean follow-up of 25.1 months. Demographic data of the overall sample are reported in Table 1. Seventy-five percent of the patients were women, and the mean age of patients was 60.8 years. According to these data, the study has a statistical power of 0.92.

**Table 1:** Demographic variables of the overall patients and stratified according to nationality and therapy

		Total sample (n = 108)			Italy (n = 58)			Finland (n = 50)		
		Overall	Local immunosuppressive therapy		Overall	Local immunosuppressive therapy		Overall	Local immunosuppressive therapy	
			No Tx	Tx		No Tx (n = 26)	Tx (n = 32)		No Tx (n = 13)	Tx (n = 37)
Age (years), mean (SD)^2^		60.8 (13.4; 17–88)	60.5 (14.6; 17–84)	61.0 (12.8; 25–88)	64.9 (12.7)	63.2 (14.8)	66.3 (10.9)	56.1 (12.8)	55.1 (13.1)	56.4 (12.8)
Gender, *N* (%)	Men	27 (25.0)	7 (17.9)	20 (29.0)	17 (29.3)	6 (23.1)	11 (34.4)	10 (20.0)	1 (7.7)	9 (24.3)
	Women	81 (75.0)	32 (82.1)	49 (71)	41 (70.7)	20 (76.9)	21 (65.6)	40 (80.0)	12 (92.3)	28 (75.7)
Follow-up, mean (SD)^4,6^		25.1 (26.6; 3–147)	15.3 (13.9; 3–68)	30.6 (30.3; 3–147	28.0 (32.6)	14.3 (14.0)	39.2 (38.8)	21.6 (16.9)	17.3 (14.1)	23.1 (17.7)
OLP clinical type, *N* (%)^1,3,7^	Atrophic/erosive form	10 (9.3)	4 (10.3)	6 (8.7)	9 (15.5)	3 (11.5)	6 (18.8)	1 (2.0)	1 (7.7)	0 (0.0)
	Plaque-like form	33 (30.6)	21 (53.8)	12 (17.4)	22 (37.9)	14 (53.8)	8 (25.0)	11 (22.0)	7 (53.8)	4 (10.8)
	Reticular/papular form	65 (60.2)	14 (35.9)	51 (73.9)	27 (46.6)	9 (34.6)	18 (56.3)	38 (76.0)	5 (38.5)	33 (89.2)
Oral medicine procedures and therapy	Annual visit, *N*	606	182	424	340	125	215	266	57	209
	Annual swab, *N*	88	20	68	37	11	26	51	9	42
	Annual biopsy, *N*^1^	102	38	64	58	30	28	44	8	36
	Annual medicines packages prescribed, *N*^1,3,5^	1735	264	1471	553	13	540	1182	251	931

^1^Statistically significant difference between overall Italian patients and Finnish patients—chi-square test *p* < 0.05

^2^Statistically significant difference between overall Italian patients and Finnish patients—ANOVA *p* < 0.05

^3^Statistically significant difference between no therapy and therapy groups in overall patients—chi-square test *p* < 0.05

^4^Statistically significant difference between no therapy and therapy groups in overall patients—ANOVA *p* < 0.05

^5^Statistically significant difference between no therapy and therapy groups in Italian patients—chi-square test *p* < 0.05

^6^Statistically significant difference between no therapy and therapy groups in Italian patients—ANOVA *p* < 0.05

^7^Statistically significant difference between no therapy and therapy groups in Finnish patients—chi-square test *p* < 0.05

^8^Statistically significant difference between no therapy and therapy groups in Finnish patients—ANOVA *p* < 0.05

Abbreviations: *Tx*, therapy (local immunosuppressive therapy); *No Tx*, no therapy (no local immunosuppressive therapy); *SD*, standard deviation; *N*, number; *OLP*, oral lichen planus

The mean annual cost was 1087.2 euros per patient. The costs were divided as follows: 593.3 euros associated with visits, 263.6 euros associated with drug therapy, 211.8 euros associated with biopsies, and 34.0 euros associated with other auxiliary tests (i.e., oral swab). Univariate analysis revealed that nationality, local immunosuppressive therapy, and clinical forms were the main variables associated with the annual costs (ANOVA *p* < 0.05) (Table 2).

**Table 2:** Summary of annual direct costs of OLP stratified according to nationality, therapy, and clinical forms

		Annual visit cost (€)^1,2,3^	Annual swab cost (€)^1,2,3,6^	Annual biopsy cost (€)^4^	Annual drug cost (€)^1,2,3,4,5,6,7^	Total annual cost (€)^1,2,3,4,5,6^
Total sample	Overall (*n* = 108)	593.3	34.0	211.8	263.6	1087.2
Local immunosuppressive therapy	Therapy group (*n* = 69)	668.8	45.6	205.3	384.7	1283.9
	No therapy group (*n* = 39)	459.6	13.6	223.2	49.3	739.1
OLP clinical type	Reticular/papular form (*n* = 65)	687.5	42.3	227.9	340.5	1275.9
	Plaque-like form (*n* = 33)	417.6	19.6	175.7	89.6	694.6
	Atrophic-erosive form (*n* = 10)	560.8	28.1	226.2	340.1	1155.6
Italy	Overall (*n* = 58)	345.6	19.6	133.2	182.3	680.7
Local immunosuppressive therapy	Therapy group (*n* = 32)	355.6	24.2	110.0	305.9	795.7
	No therapy group (*n* = 26)	333.2	13.9	161.8	30.2	539.1
OLP clinical type	Reticular/papular form (*n* = 21)	330.0	17.3	125.3	189.9	662.5
	Plaque-like form (*n* = 21)	305.1	17.7	132.5	92.7	548.0
	Atrophic-erosive form (*n* = 16)	491.1	31.3	158.9	378.3	1059.5
Finland	Overall (*n* = 50)	880.6	50.8	302.9	357.9	1558.7
Local immunosuppressive therapy	Therapy group (*n* = 37)	939.7	64.0	287.7	452.9	1706.2
	No therapy group (*n* = 13)	712.5	13.0	345.9	87.4	1139.0
OLP clinical type	Reticular form (*n* = 38)	941.4	60.0	300.7	446.8	1711.8
	Plaque form (*n* = 11)	642.6	23.4	262.1	83.3	988.0
	Atrophic-erosive form (*n* = 1)	1188.0	0.0	832.0	0.0	2020.0

^1^Statistically significant difference between therapy and no therapy groups in overall patients—ANOVA *p* < 0.05

^2^Statistically significant difference between clinical forms in overall patients—ANOVA *p* < 0.05

^3^Statistically significant difference between overall Italian patients and Finnish patients—ANOVA *p* < 0.05

^4^Statistically significant difference between therapy and no therapy groups in Italian patients—ANOVA *p* < 0.05

^5^Statistically significant difference between clinical forms in Italian patients—ANOVA *p* < 0.05

^6^Statistically significant difference between therapy and no therapy groups in Finnish patients—ANOVA *p* < 0.05

^7^Statistically significant difference between clinical forms in Finnish patients—ANOVA *p* < 0.05

Abbreviations: *OLP*, oral lichen planus; *n*, number

Due to differences between Italian and Finnish cohort (Table 1) and their public health care system, a further analysis was performed for each center.

Among the Italian cohort, 58 Italian patients (41 women and 17 men, mean age of 64.9 years) were included in this study with a mean follow-up of 28.0 months. The demographic data of the Italian sample are reported in Table 1. The average annual cost is € 680.7 per patient. Univariate analysis revealed that local immunosuppressive therapy and clinical forms were the variables associated with the annual costs (ANOVA *p* < 0.05) (Table 2). Moreover, the atrophic and the erosive forms represent the variable associated with higher costs in the group of patients undergoing local immunosuppressive therapy; no differences were noted between the clinical forms in patients who did not receive local immunosuppressive therapy (Table 3).

**Table 3:** Summary of annual direct costs of OLP stratified according to therapy

		Annual visit cost (€)	Annual swab cost (€)^3^	Annual biopsy cost (€)^1^	Annual drug cost (€)^1,2,3^	Total annual cost (€)^1,2,3^
Italy	Overall (*n* = 58)	345.6	19.6	133.2	182.3	680.7
Therapy group (*n* = 32)		355.6	24.2	110.0	305.9	795.7
	Reticular/papular form (*n* = 18)	312.7	19.4	99.7	252.1	684.0
	Plaque-like form (*n* = 8)	282.9	17.9	91.7	230.6	623.1
	Atrophic-erosive form (*n* = 6)	581.4	46.9	165.5	567.5	1361.2
No therapy group (*n* = 26)		333.2	13.9	161.8	30.2	539.1
	Reticular/papular form (*n* = 9)	364.6	12.9	176.5	65.6	619.6
	Plaque-like form (*n* = 14)	317.8	17.6	155.8	13.9	505.1
	Atrophic-erosive form (*n* = 3)	310.5	0.0	145.6	0.0	456.2
Finland	Overall (*n* = 50)	880.6	50.8	302.9	357.9	1558.7
Therapy group (*n* = 37)		939.7	64.0	287.7	452.9	1706.2
	Reticular/papular form (*n* = 33)	981.7	66.5	298.5	480.0	1783.9
	Plaque-like form (*n* = 4)	593.3	43.8	198.8	229.0	1064.9
	Atrophic-erosive form (*n* = 0)	-	-	-	-	-
No therapy group (*n* = 13)		712.5	13.0	345.9	87.4	1139.0
	Reticular/papular form (*n* = 5)	675.9	17.4	315.4	227.2	1235.9
	Plaque-like form (*n* = 7)	670.8	11.7	298.3	0.0	944.0
	Atrophic-erosive form (*n* = 1)	1188.0	0.0	832.0	0.0	2020.0

^1^Statistically significant difference between therapy and no therapy groups in Italian patients—ANOVA *p* < 0.05

^2^Statistically significant difference between clinical forms in the Italian therapy group—ANOVA *p* < 0.05

^3^Statistically significant difference between therapy and no therapy groups in Finnish patients—ANOVA *p* < 0.05

Abbreviations: *OLP*, oral lichen planus; *n*, number

Among the Finnish cohort, 50 patients (40 women and 10 men, mean age 56.1) were included with a mean follow-up of 21.6 months. The demographic data of the Finnish sample are reported in Table 1. The average annual cost is € 1558.7 per patient. Univariate analysis revealed that local immunosuppressive therapy and clinical forms were the variables associated with the annual costs (ANOVA *p* < 0.05) (Table 2). Subgroup analysis according to therapy revealed no differences between clinical forms (Table 3).

## Discussion

The continuous aging of the population and the growing number of chronic diseases represent one of the main challenges of the twenty-first century, with important repercussions on the strategic choices of public health care. In this perspective, cost of illness (COI) study is an excellent tool to provide an economic framework useful for ensuring an effective allocation of public health resources [10]. Although oral health is one of the most common public health problems, few studies have evaluated the impact in economic terms of the main chronic diseases of the oral cavity (dental caries, periodontal disease, oral cancer, and oral potentially malignant disorders). From the analysis of the literature, only one study has been previously conducted aimed at estimating the direct health costs of a cohort of patients with OLP, from the time of diagnosis until the last consultation. The study conducted by Ni Riordain et al. [11] in a cohort of 100 English patients (30 men and 70 women) identified a direct annual cost of £ 398.58 (€ 541.16) per patient, which greatly differs from the identified annual costs in our study (annual cost per person of 1087.2 € per person). Ni Riordain et al. also stratified the costs in relation to the severity of the disease based on the therapeutic protocol (only local or combined with systemic therapy), reporting a higher cost for patients who needed systemic therapy (£ 663 (€ 900.16) vs. £ 301 (€ 408.73)), in a similar way to what was found in our study. The important cost difference compared to our study (average of € 541 vs. € 1087) must take into account some methodological differences: first, the study by Ni Riordain et al. [11] was conducted by administering questionnaires, primarily aimed at identifying the number of examinations, by oral medicine doctors and by GPs, directly to patients, and any drugs prescribed for the treatment of OLP during the last 12 months. The study did not analyze the medical records, which should be more realistic than a questionnaire (recall bias). Secondly, the study conducted by Ni Riordain et al. [11] represents a retrospective 12-month pilot cost study, while our study estimated the costs incurred during a longer follow-up (about 24 months). Considering the results deriving from the whole population studied (Italian and Finnish cohorts), it was possible to notice an increase in costs, in relation to the need to prescribe pharmacological therapy for OLP. Drug therapy, corticosteroids or other immunosuppressive drugs, covers about 1/4 of the annual costs (€ 263, 24% of the total) considering the entire sample studied. Specifically, comparing the cost of patients who did not get local immunosuppressive therapy with those who did, the average annual costs increase significantly from € 539 to € 796 in Italy and from 1139 € to 1706 € in Finland (*p* < 0.05) (Table 2). A considerable part of drug therapy–related costs also refers to topical antifungals to treat mycotic overinfections and to swabs performed to diagnose it, due to immunosuppressive therapy cycles, both local and systemic.

In the overall calculation (Table 2), the annual costs associated with outpatient examinations (593.3 €) and diagnostic procedures (biopsies and swabs—211.8 € and 34.0 €, respectively) correspond to little more than 2/3 (77.2%) of the annual expenditure: the checkups are needed both for the chronic nature of OLP and for the possibility of malignant transformation. Since no specific guidelines are available for the follow-up of these patients, the clinicians sometimes feel insecure about the course of the disease and thus prefer a strict follow-up. Mignogna et al. [12] stressed the importance of a regular follow-up, at least three times a year. If the clinical examination suggests disease progression, the follow-up period must be halved to 2 months. OLP clinical manifestation poses many diagnostic challenges with other disorders (i.e., verrucous-proliferative leukoplakia, bullous diseases, candidiasis, erythroplakia, lichenoid lesions), and biopsy is essential to achieve a final diagnose [9]. Furthermore, even during the follow-up, more biopsies can be indicated when an alteration of the clinical features is observed [13]. Our previous systematic review highlights a malignant transformation rate of 1.4% and confirms that OLP is a potentially malignant disorder [5]. In general, the potentially malignant nature of the disease and the features of the lesions might require multiple and repeated biopsies over time [12, 14], thus increasing the direct costs of the OLP. In our study, 4 out of 108 OLP cases developed an oral squamous cell carcinoma (OSCC) in a 2-year mean follow-up, so the overall transformation rate in our sample was similar to that reported in literature [5]: one patient had a concomitant diagnosis of OSCC at the diagnosis of OLP and the other three patients at 12 months, 37 months, and 120 months after OLP diagnosis. All 4 cases of malignant transformation were diagnosed at early stage (stage 1), and this resulted in an increase of the 5-year chance of survival, a better quality of life, and a significant reduction in the costs associated with the cancer management. Considering the follow-up of OLP patients, it is fundamental to give patients the most appropriate treatment, especially when symptomatic, and to perform early diagnosis if malignant transformation is suspected.

The multicenter design of the study allows for a series of observations between the Finnish and Italian cohorts. A first observation is of economic nature: this study provides a detailed economic frame for each center, considering that they belong to different public health care systems. The Italian one provides coverage for all citizens and supports, through a regional health care system, almost all medical expenses, completely free or “co-paid” by the patient, depending on the disease, on the wealth of the patient, and on other reasons (e.g., physical or mental disability, rare pathologies). People with OLP are not exempted from co-pay; therefore, medical costs are partly borne by the Italian public health care system and partly by the patients. In Finland, health care is mainly financed and provided by the government. Municipalities represent local authorities and are responsible for organizing all health care services to their residents. The financing of health care services is mainly based on public funding and to a small extent co-paid by patients. The annual expenses related to OLP are more expensive in the Finnish cohort (€ 1558.7) than in the Italian one (€ 680.7) (*p* < 0.05): this result could be due to the different therapeutic management (e.g., more patients in the Finnish cohort received immunosuppressive therapy—*p* < 0.05) (Table 1) and the difference between the Italian and Finnish cost of drugs and health services. Furthermore, a difference between Finland and Italy was evident between the percentage of individuals receiving local immunosuppressive therapy (74% vs. 55%, respectively—chi-squared test *p* < 0.05). This percentage is particularly high in Finland: probably the most complex cases are treated in a reference center, while simpler ones could be managed by general practitioners or in smaller health care centers. To this regard, in the Finnish cohort, a higher percentage of subjects with reticular form received local immunosuppressive therapy when compared with the Italian, causing a considerable increase in the costs of the reticular forms of the Finnish cohort (Table 3). Moreover, only one erosive case was reported in Finnish cohort, which could represent a bias for the determination of costs (Table 3). Considering the Italian cohort, the percentage of subjects in therapy is in line with the data reported in the literature (50% of subjects are symptomatic) [15]. Nevertheless, the difference between Italian and Finnish costs should be analyzed in the light of the different purchasing power, living costs, and average population income.

Another consideration refers to the different therapeutic approaches between Italy and Finland: since there are no stringent guidelines regarding OLP therapy, there are various medications available on the market in different formulations. In the Italian study population, the most used symptomatic drug therapy is based on clobetasol propionate available as an ointment, sometimes in association with rinses with azoles or nystatin, whereas the Kuopio University Hospital adopts a variety of different therapeutic regimens (Table 4). Furthermore, the absence of strict indications on the amount of drug to be applied made it difficult to identify actual consumption and, therefore, we relied on an (over)estimate of about 5 g of drug per single application. Comparing the expenses incurred by individuals in immunosuppressive local therapy vs. those not in therapy, Finnish costs related to swabs were significantly higher in the “therapy” group (*p* < 0.05), while in Italy, costs connected to biopsies were higher in the group undergoing therapy (*p* < 0.05). The follow-up was different between these two groups, being longer in the symptomatic subjects under therapy (*p* < 0.05 both in the overall population and in the Italian study population). In the Finnish study population, follow-up was not statistically different among the two groups and this could be explained by a reduced average follow-up time of the Finnish cohort. To this regard, in Finland, but not in Italy, phone call checkups are often used to monitor the clinical course of patients, especially to investigate the effects of the treatment on symptoms. Nevertheless, the cost related to phone call was not included in the cost analysis.

**Table 4:** Drug prescription frequency stratified according to nationality

Drug prescription		Overall (*n* = 3139)	Italy (*n* = 1653)	Finland (*n* = 1486)
Local immunosuppressive therapy, *N* (%)	Clobetasol	876 (63.2)	865 (96.3)	11 (2.2)
	Cyclosporin	7 (0.5)	7 (0.8)	0 (0.0)
	Fluocinonide	14 (1.0)	14 (1.6)	0 (0.0)
	Betamethasone	113 (8.1)	0 (0.0)	113 (23.1)
	Hydrocortisone	2 (0.1)	0 (0.0)	2 (0.4)
	Triamcinolone acetonide	145 (10.5)	0 (0.0)	145 (29.7)
	3 gel (nystatin, lidocaine, chlorhexidine	10 (0.7)	0 (0.0)	10 (2.0)
	Tacrolimus	219 (15.8)	12 (1.3)	207 (42.3)
	Beclomethasone dipropionate	1 (0.1)	0 (0.0)	1 (0.2)
Local antimycotic therapy, *N* (%)	Nystatin	1063 (61.0)	108 (14.4)	955 (96.2)
	Fluconazole	641 (36.8)	641 (85.6)	0 (0.0)
	Miconazole	9 (0.8)	0 (0.0)	9 (0.9)
	Clotrimazole	23 (1.3)	0 (0.0)	23 (2.3)
	Amphotericin B	6 (0.3)	0 (0.0)	6 (0.6)
Systemic immunosuppressive therapy, *N* (%)	Deltacortene	3 (75.0)	3 (100.0)	0
	Prednisolone	1 (25.0)	0 (0.0)	1 (100.0)
Systemic antimycotic therapy, *N* (%)	Fluconazole	5 (83.3)	3 (100.0)	2 (66.7)
	Itraconazole	1 (16.7)	0 (0.0)	1 (33.3)

Abbreviations: *N*, number

A further consideration concerns the role of the clinical type of OLP in the determination of direct costs. Some clinical forms (i.e., atrophic and erosive forms) are associated with a greater severity of the disease and, frequently, are symptomatic, thus requiring immunosuppressive therapy. To this regard, differences in annual costs were retrieved between the main clinical forms, in both the Italian and Finnish population at univariate analysis, being the atrophic and the erosive forms most expensive (Table 2). Furthermore, within the group of patients who receive local therapy, in the Italian cohort, the atrophic and erosive forms were any way the most expensive form (Table 3).

In the literature, few studies have been conducted to evaluate the costs of chronic oral pathologies: in a study of the costs associated with periodontal disease conducted in Malaysia, the authors reported the direct cost of € 678, being very similar to the costs of the OLP observed in this study [16]. However, this estimate concerns only the first year of periodontal therapy which represents the most intense phase of therapy and which requires several medical and surgical examinations and procedures.

The limitations of our study lie in the design of the study itself: the retrospective nature of the study did not make it possible to count direct non-health care costs and indirect costs. The multicenter design of the study reflects the situation of two tertiary oral medicine/head and neck clinics, where probably the most severe or symptomatic OLP cases are evaluated and treated, which in turn may inflate the costs associated with pharmacological therapies. In addition, patient follow-up varies widely and, in some cases, does not reach the first year of observation, partly causing an overestimation of the annual costs. On the other hand, patients with a longer follow-up may have led to an underestimation of costs since after the first year, when it is not unusual to adopt a less invasive diagnostic and therapeutic approach. Further studies could investigate whether there are differences between the first year of treatment and subsequent years in relation to the course of the disease, and multicenter national studies should be encouraged in order to understand regional differences. Furthermore, an aspect that has not been possible to investigate is the patient’s true compliance to the therapy, whether the prescribed therapeutic regimen has been adhered to without abusing or reducing the amount of drug to be applied. Due to the retrospective nature of this study, it was not possible to differentiate patients affected by erosive OLP from those affected by atrophic form, among which erosive forms could undergo therapy more frequently. Moreover, it was not possible to evaluate costs related to other laboratory procedures as blood exams and ancillary laboratory techniques (e.g., immunohistochemistry).

## Conclusion

In this multicenter COI study of OLP estimating the direct health care costs, we found that the costs are very similar to other chronic oral disorders (i.e., periodontitis) and that patients with more severe clinical features, who therefore need symptomatic therapy, are facing more expensive costs. Outpatient examinations represent the most important part of the costs incurred, according to the chronic nature of the disease. Furthermore, these results provide strong evidence of the different therapeutic approaches adopted by the two hospitals in the absence of stringent guidelines. Further studies are therefore needed to quantify other components of the costs associated with lichen (direct non-health care costs and indirect costs) for an overall analysis of the economic impact due to the disease.
